# Amyloid-β peptides in cerebrospinal fluid of patients with dementia with Lewy bodies

**DOI:** 10.1186/s13195-019-0537-5

**Published:** 2019-10-10

**Authors:** Inger van Steenoven, Wiesje M. van der Flier, Philip Scheltens, Charlotte E. Teunissen, Afina W. Lemstra

**Affiliations:** 1 Alzheimer Center Amsterdam, Department of Neurology, Amsterdam Neuroscience, Vrije Universiteit Amsterdam, Amsterdam UMC, De Boelelaan 1118, 1081 HV Amsterdam, The Netherlands; 2 Department of Epidemiology and Biostatistics, Amsterdam Neuroscience, Vrije Universiteit Amsterdam, Amsterdam UMC, Amsterdam, The Netherlands; 3 Neurochemistry Laboratory and Biobank, Department of Clinical Chemistry, Amsterdam Neuroscience, Vrije Universiteit Amsterdam, Amsterdam UMC, Amsterdam, The Netherlands

**Keywords:** Cerebrospinal fluid, Dementia with Lewy bodies, Amyloid-β peptides, Co-morbid Alzheimer’s disease pathology

## Abstract

**Background:**

One of the major challenges in diagnosing dementia with Lewy bodies (DLB) is the common co-morbid presence of amyloid pathology. To understand the putative role of altered amyloid-β (Aβ) metabolism in dementia with DLB, we analyzed levels of different cerebrospinal fluid (CSF) Aβ peptides (Aβ38, Aβ40, Aβ42) in DLB, Alzheimer’s disease (AD), and cognitively normal controls.

**Methods:**

CSF from patients with DLB (*n* = 72; age 68 ± 6 years; 10%F; Mini-mental State examination (MMSE) 23 ± 4), AD (*n* = 38; age 68 ± 6 years; 8%F; MMSE 22 ± 5), and cognitively normal controls (*n* = 38; age 67 ± 7 years; 13%F; MMSE 29 ± 2) was analyzed using the Meso Scale Discovery assay for human Aβ peptides. We performed general linear models to compare CSF Aβ peptide levels between groups. Associations between CSF Aβ peptides and MMSE score at baseline and longitudinal changes over time were assessed with linear mixed models.

**Results:**

For all three CSF Aβ peptides and compared to controls (Aβ38 2676 ± 703 pg/ml, Aβ40 6243 ± 1500 pg/ml, and Aβ42 692 ± 205 pg/ml), we observed lower levels in DLB (Aβ38 2247 ± 638, Aβ40 5432 ± 1340, and Aβ42 441 ± 185, *p* < 0.05), whereas AD patients showed only lower Aβ42 levels (304 ± 71, *p* < 0.001). The observed differences in Aβ38 and Aβ40 were independent of co-morbid AD pathology (CSF tau/Aβ42 > 0.52) and APOE genotype. Finally, lower Aβ peptide levels were associated with lower MMSE score (*β* = 1.02–1.11, *p* < 0.05).

**Conclusion:**

We demonstrated different profiles of CSF Aβ reduction in DLB and AD. In particular, while AD is characterized by an isolated drop in Aβ42, DLB comes with reductions in Aβ38, Aβ40, and Aβ42. This suggests that amyloid metabolism is affected in DLB, even in the absence of co-morbid AD pathology.

## Background

Dementia with Lewy bodies (DLB) is the second most common neurodegenerative disease in the elderly after Alzheimer’s disease (AD), accounting for up to 20% of the dementia cases [1]. Next to dementia, core features of DLB are parkinsonism, visual hallucinations, fluctuations in cognition and attention, and rapid eye movement (REM) sleep behavior disorder (RBD) [2]_._ DLB is characterized neuropathologically by the accumulation of α-synuclein aggregates in Lewy bodies and Lewy neurites throughout the brain [3]. In addition, DLB patients often have some degree of concomitant AD-related pathology, i.e., extracellular amyloid-β (Aβ) aggregation and intracellular tau deposition, such that up to 50% of DLB patients have a high-level AD pathology [4, 5] and approximately 25% have an AD profile in cerebrospinal fluid (CSF) [6]. Several studies demonstrated that co-morbid AD pathology influences clinical diagnostic accuracy [7] and is related with a more severe disease course in DLB [5, 8–10]. However, the pathophysiological processes underlying the co-existence of AD pathology in DLB are still unknown.

Several lines of evidence suggest that an imbalance between production and clearance of Aβ is the initiating factor that contributes to AD pathology [11, 12]. Aβ is a proteolytic cleavage product of amyloid precursor protein (APP). In the amyloidogenic pathway, cleavage by β- and γ-secretase results in different Aβ peptides, ranging from 38 to 43 amino acids. The precise location and the number of cleavages determine the ultimate length of the Aβ peptide [13]. The most abundant Aβ peptides in CSF are Aβ38, Aβ40, and Aβ42 [14]. CSF Aβ peptide levels might reflect to some extent the dysregulation of Aβ metabolism (production and clearance) and Aβ aggregation in the brain. Aβ42 is prone to deposition in amyloid plaques. Reduced levels of Aβ42 in CSF are thought to reflect Aβ42 sequestration in amyloid plaques in the brain [15, 16]. The shorter Aβ peptides, Aβ38 and Aβ40, are less prone to aggregate and their CSF concentrations rather reflect production of Aβ peptides from APP by β- and γ-secretases [17]. In contrast with AD, reduced CSF levels of all three Aβ peptides in DLB have been reported in studies with small numbers of included DLB patients [18–23]. However, CSF Aβ peptides have not yet been validated in large, well-characterized clinical cohorts, and none of these studies addressed the issue of co-morbid AD pathology in DLB.

In the present study, we aim to (1) characterize the levels of three different CSF Aβ peptides (Aβ42, Aβ40, and Aβ38) in DLB and compare this with levels in AD and cognitively normal controls and (2) investigate whether evidence of AD pathology defined by a CSF profile compatible with AD influences CSF levels of Aβ peptides in DLB patients. Finally, we studied whether specific CSF Aβ peptides were associated with cognitive decline in DLB.

## Methods

### Study population

**Fig. 1:**
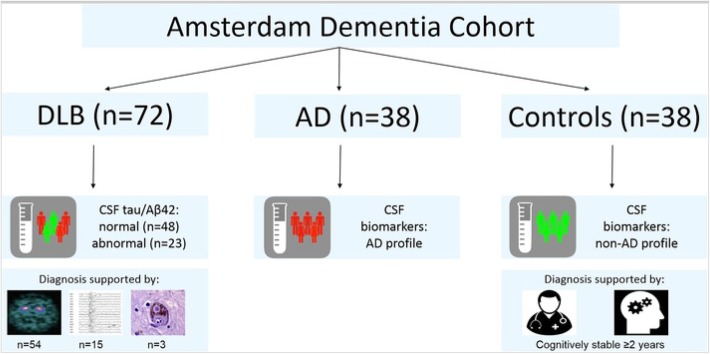
Flow chart of patient selection. We selected 72 patients with a diagnosis of probable DLB and matched them for age and sex with 38 patients with a diagnosis of probable AD and 38 patients with subjective cognitive decline (SCD) who served as control subjects from the Amsterdam Dementia Cohort. DLB patients were stratified into two groups: DLB patients with an AD CSF profile (CSF tau/Aβ_1-42_ ≥ 0.52 [24]; DLB AD+, *n* = 23) and DLB patients with a normal CSF profile (CSF tau/Aβ_1-42_ < 0.52; DLB AD−, *n* = 48). The diagnosis of DLB was supported by (123) I-FP-CIT-SPECT (DAT-SPECT) findings showing presynaptic dopaminergic deficits (*n* = 54, 75%) or by slow-wave activity on EEG (*n* = 15, 21%), or was confirmed at autopsy (*n* = 3, 4%). All AD patients had a CSF profile compatible with AD. All controls had normal AD biomarker levels and preserved normal cognitive function on neuropsychological testing for at least 2 years after first presentation at the memory clinic

We included 72 patients with a diagnosis of probable DLB and matched them for age and sex with 38 patients with a diagnosis of probable AD and 38 subjects with subjective cognitive decline (SCD) who served as controls (Fig. 1). The abovementioned patients and controls were selected from the Amsterdam Dementia Cohort [25], consisting of patients who were assessed at the Alzheimer Center Amsterdam between January 2000 and December 2017, based on the availability of CSF. All selected patients and controls underwent an extensive standardized and multidisciplinary workup, as part of the routine clinical practice, including medical history, physical and neurological examinations, neuropsychological evaluation, electroencephalography (EEG), brain magnetic resonance imaging (MRI), and laboratory tests including lumbar puncture and apolipoprotein E (APOE) genotyping. Biomaterial is available for 67% of all patients in the Amsterdam Dementia Cohort [25]. Diagnoses were made in a multidisciplinary consensus meeting. DLB was diagnosed according to the clinical diagnostic consensus criteria for probable DLB [2]. The diagnosis of DLB was supported by (123) I-FP-CIT-SPECT (DAT-SPECT) findings showing presynaptic dopaminergic deficits (*n* = 54, 75%) or by slow-wave activity on EEG (*n* = 15, 21%), or was confirmed at autopsy (*n* = 3, 4%). Patients with AD were diagnosed according to the National Institute for Neurological and Communicative Diseases AD and Related Disorders Association (NIA-AAA) criteria for probable AD [26], with probability of AD etiology based on the AD CSF biomarkers. Subjects were labeled as SCD when no abnormalities on clinical or cognitive testing were observed and the criteria for MCI, dementia, or other medical conditions potentially causing cognitive decline were not met. To be included as controls in the current study, they had to fulfill also the following additional inclusion criteria: (1) normal AD biomarker levels and (2) preserved normal cognitive function on neuropsychological testing for at least 2 years after the first presentation at the memory clinic. The study was approved by the local medical ethics committee, and all subjects gave their written informed consent for the use of their clinical data and CSF for research purposes.

### Standard CSF procedures

In line with the international biobanking consensus guidelines [27], CSF was obtained by lumbar puncture using a 25-gauge needle and a syringe and collected into 10-mL polypropylene tubes (Sarstedt, Nümbrecht, Germany). Part of the CSF was used for routine analysis, including leukocyte and erythrocyte count, glucose concentration, total protein concentration, and Aβ_1-42_, total tau, and p-tau concentrations (Innotest®, Fujirebio, Gent, Belgium). The ratio of CSF total tau and Aβ_1-42_, measured with Innotest enzyme immunoassay, was used to determine the presence of an AD profile in CSF (CSF tau/Aβ_1-42_ ≥ 0.52 [24]). Within 2 h, 2 ml CSF was centrifuged at 1800×*g* for 10 min at 4 °C, transferred to new polypropylene tubes, and stored at − 20 °C for routine biomarker analysis. The remaining CSF was processed similarly, but stored directly at − 80 °C for biobanking.

### Measurement of CSF Aβ38, Aβ40, and Aβ42

For this study, CSF Aβ42, Aβ40, and Aβ38 concentrations were determined with Meso Scale Discovery (MSD) Abeta 3-Plex Kit (Meso Scale Diagnostic, Rockville, USA).

### Cognitive follow-up

Follow-up for all patients took place by annual routine visits to the memory clinic in which physical and neurological examination and cognitive assessment were repeated. Each DLB patient underwent at least one cognitive assessment. Follow-up MMSE data were available in 51 (71%) DLB patients, with a mean follow-up time of 2.7 ± 1.8 years. Follow-up extended up to 8 years for individual patients.

### Statistical analyses

Analyses were performed using R (version 3.2.5, R Development Core Team 2010). To assess group differences at baseline, univariate analysis of variance (ANOVA), *χ*^2^, and Kruskal-Wallis *H* tests were performed where appropriate. Differences in CSF Aβ peptide levels between groups were compared using ANOVA corrected for age and sex in conjunction with Student *t* tests corrected for multiple comparisons using a false discovery rate (FDR) correction. We assessed associations of the CSF Aβ peptides using Pearson correlations. Results were corrected for multiple comparisons using FDR correction.

Associations between CSF Aβ peptides and MMSE score at baseline and longitudinal changes over time were assessed with linear mixed models. The models included terms for time (years), CSF Aβ peptide measures, and an interaction term of CSF Aβ peptide measure × time as independent variables and MMSE score as the dependent variable and were adjusted for age, sex, and education. For all models, a random intercept and slope were assumed. Aβ peptide levels were transformed to *z*-scores. A beta coefficient of 1 (*β* = 1) therefore implies that a 1 standard deviation increase in CSF Aβ peptide was associated with a 1-point increase in MMSE score. A *p* value < 0.05 was considered significant.

## Results

### Patient characteristics

Table 1 displays the demographics, clinical characteristics, and CSF biomarker characteristics per diagnostic group. Diagnostic groups had similar age and sex distribution, showing effective matching. Dementia patients (DLB and AD) showed lower MMSE scores at baseline compared to controls (*p* < 0.001). There were more APOEε4 carriers in DLB and AD groups compared to controls (*p* < 0.05).

**Table 1:** Demographics, clinical characteristics, and CSF biomarker characteristics in DLB, AD, and controls

	DLB (*n* = 72)	AD (*n* = 38)	Controls (*n* = 38)
Female (*n*, %)	7 (10%)	3 (8%)	5 (13%)
Age (mean ± SD)	68 ± 6	68 ± 6	67 ± 6
MMSE (median [IQR])	23 [21–26]^b^	22 [18–25]^b^	29 [28–30]
APOEε4 carrier (*n*, %)	39 (57%)^a^	26 (72%)^a^	12 (32%)
CSF AD biomarkers Innotest (median [IQR])			
Aβ_1-42_ (pg/ml)*	790 [638–1040]^b,d^	620 [562–660]^b^	1123 [1022–1291]
t-tau (pg/ml)	306 [228–368]^b,d^	611 [498–791]^b^	230 [187–271]
p-tau (pg/ml)	47 [35–60]^d^	79 [65–99]^b^	44 [34–50]
CSF tau/Aβ42 > 0.52 (*n*, %)	23 (33%)^b,d^	38 (100%)^b^	0 (0%)
CSF Aβ peptides MSD (mean ± SD)			
Aβ42 (pg/ml)	441 ± 185^b,d^	304 ± 71^b^	692 ± 205
Aβ40 (pg/ml)	5432 ± 1340^a^	5897 ± 1066	6243 ± 1500
Aβ38 (pg/ml)	2247 ± 638^a,c^	2524 ± 547	2676 ± 703
Aβ42/Aβ40 ratio	0.08 ± 0.03^b,d^	0.05 ± 0.01^b^	0.11 ± 0.02
Aβ42/Aβ38 ratio	0.20 ± 0.07^b,d^	0.12 ± 0.03^b^	0.26 ± 0.04
Aβ38/Aβ40 ratio	0.41 ± 0.03^a,c^	0.43 ± 0.04	0.43 ± 0.02

Data are presented as mean ± SD, median [interquartile range], or *n* (%). Differences between groups were assessed with ANOVA, *χ*^2^, and Kruskal-Wallis *H* tests where appropriate. For CSFAβ peptides, differences between diagnostic groups were assessed using ANOVA corrected for multiple comparisons using a false discovery rate (FDR) correction

*Abbreviations: Aβ42* amyloid β_1-42_ determined with MSD ELISA assay, *Aβ40* amyloid β_1-40_ determined with MSD ELISA assay, Aβ38 amyloid β_1-38_ determined with MSD ELISA assay, AD Alzheimer’s disease, *DLB* dementia with Lewy bodies, MMSE mini-mental state examination, *MSD* Meso Scale Discovery

^a^*p* < 0.05 compared to controls; ^b^*p* < 0.001 compared to controls; ^c^*p* < 0.05 compared to AD; ^d^*p* < 0.001 compared to AD

*Levels of Innotest Aβ_1-42_ were drift corrected [28]

### CSF Aβ peptides in DLB, AD, and controls

**Fig. 2:**
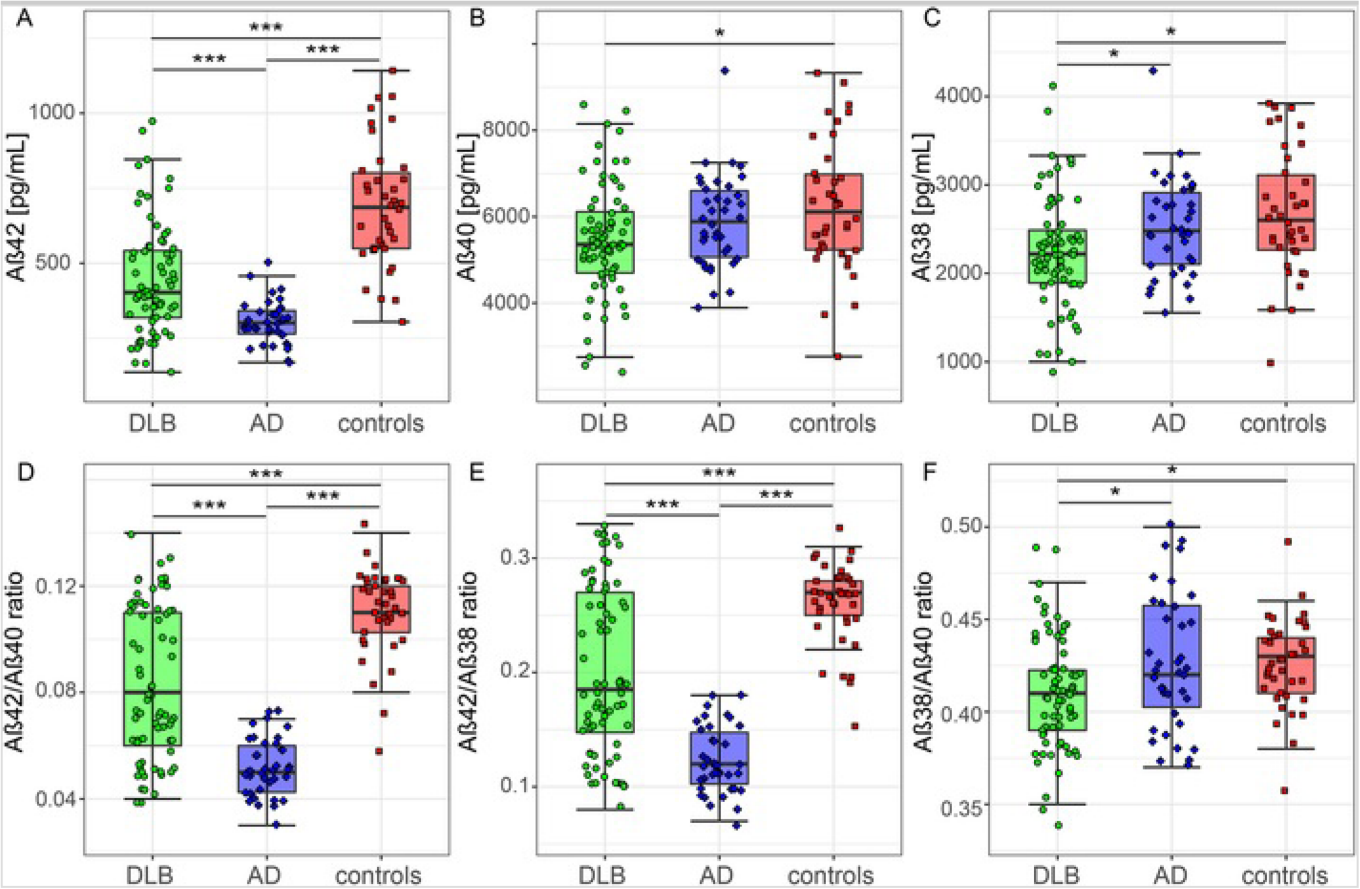
CSF Aβ peptides in DLB, AD, and controls*.* **a** CSF levels of Aβ42. **b** CSF levels of Aβ40. **c** CSF levels of Aβ38. **d** CSF Aβ42/Aβ40 ratio. **e** CSF Aβ42/Aβ38 ratio. **f** CSF Aβ38/Aβ40 ratio. The line through the middle of the boxes corresponds to the median and the lower and the upper lines to the 25th and 75th percentile, respectively. The whiskers extend from the 5th percentile on the bottom to the 95th percentile on the top. Differences between groups were assessed with ANOVA with FDR multiple comparison correction. *p < 0.05, **p < 0.01, ***p < 0.001

DLB patients had lower CSF levels of all three Aβ peptides (Aβ38 2247 ± 638 pg/ml, Aβ40 5432 ± 1340 pg/ml, Aβ42 441 ± 185 pg/ml) compared to controls (*p* < 0.05), whereas AD patients showed only lower levels of Aβ42 (304 ± 71 pg/ml) compared to controls (*p* < 0.001). Moreover, DLB patients had lower levels of Aβ38 as compared with AD (*p* < 0.05, Table 1 and Fig. 2a–c). Consequently, the ratios Aβ42/Aβ40 and Aβ42/Aβ38 were the lowest in AD and the highest in controls, and DLB patients had values in between (*p* < 0.001 compared to AD and controls). Aβ38/Aβ40 ratio was the lowest in DLB (*p* < 0.05 compared to AD and controls, Table 1 and Fig. 2d–f). Associations between Aβ peptides in CSF are shown in Additional file 1: Table S1. Throughout all investigated diagnostic groups, the different Aβ peptide levels were strongly positively correlated to each other (all *r* > 0.5), especially the correlation between Aβ40 and Aβ38 was strong (0.93 < *r* < .98, *p* < 0.001). In AD and DLB patients, the Aβ42/Aβ40 and Aβ42/Aβ38 ratios were inversely associated with the ratio between tau and Aβ42 (− 0.5 < *r* < − 0.71, *p* < 0.05). In controls, however, no associations were found. In AD, but not in DLB, the Aβ38/Aβ40 ratio correlated positively with the tau/Aβ42 ratio (*r* = 0.52, *p* < 0.05). No associations were found between any of the Aβ peptides and age and sex.

### CSF Aβ peptides in DLB subgroups

**Fig. 3:**
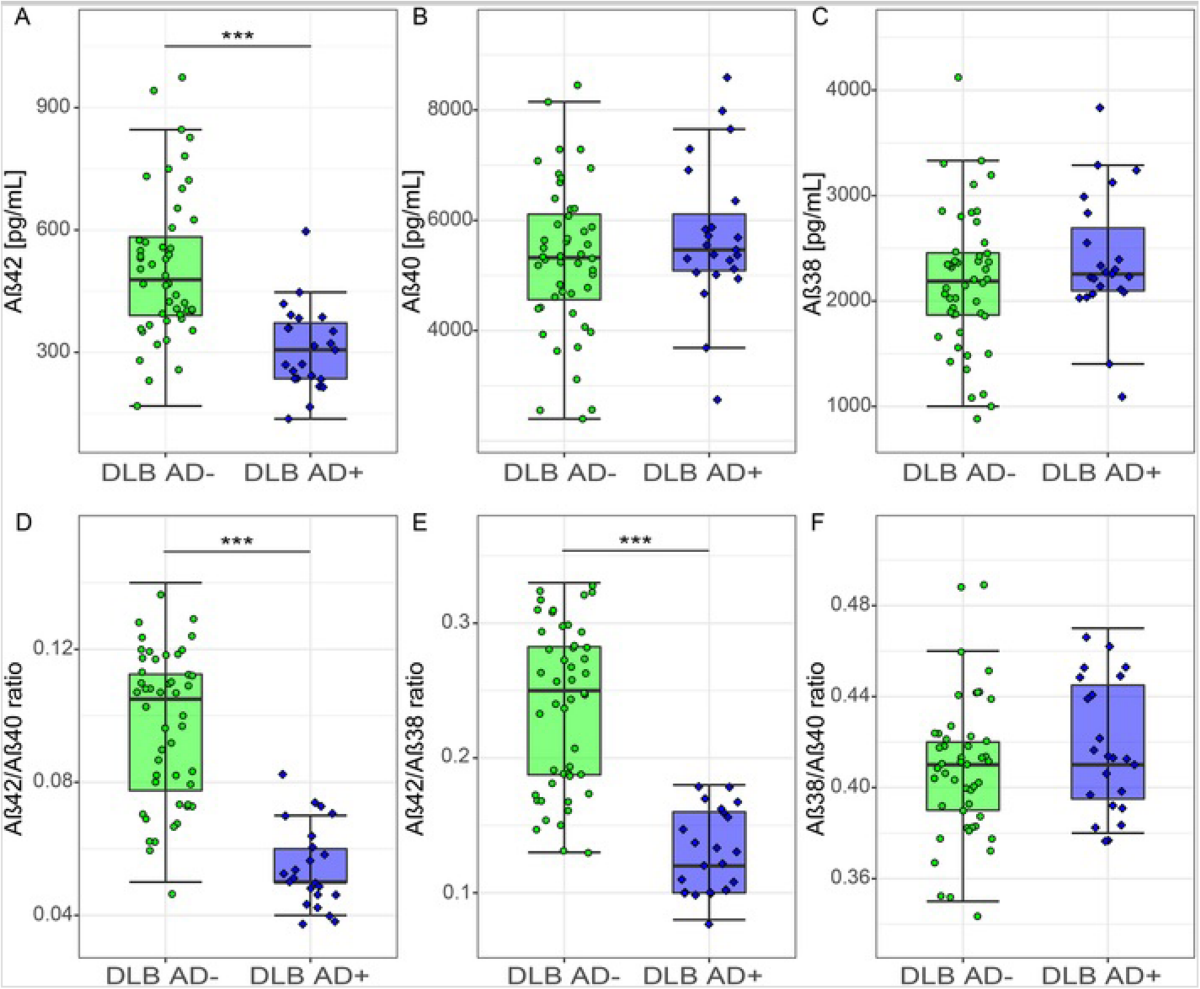
CSF Aβ peptides stratified by CSF tau/Aβ42 ratio in DLB. **a** CSF levels of Aβ42. **b** CSF levels of Aβ40. **c** CSF levels of Aβ38. **d** CSF Aβ42/Aβ40 ratio. **e** CSF Aβ42/Aβ38 ratio. **f** CSF Aβ38/Aβ40 ratio. The line through the middle of the boxes corresponds to the median and the lower and the upper lines to the 25th and 75th percentile, respectively. The whiskers extend from the 5th percentile on the bottom to the 95th percentile on the top. For visualization purposes, the AD and control groups are also presented. Differences between DLB AD− and DLB AD+ were assessed with ANOVA corrected for age and sex. *p < 0.05, **p < 0.01, ***p < 0.001

**Fig. 4:**
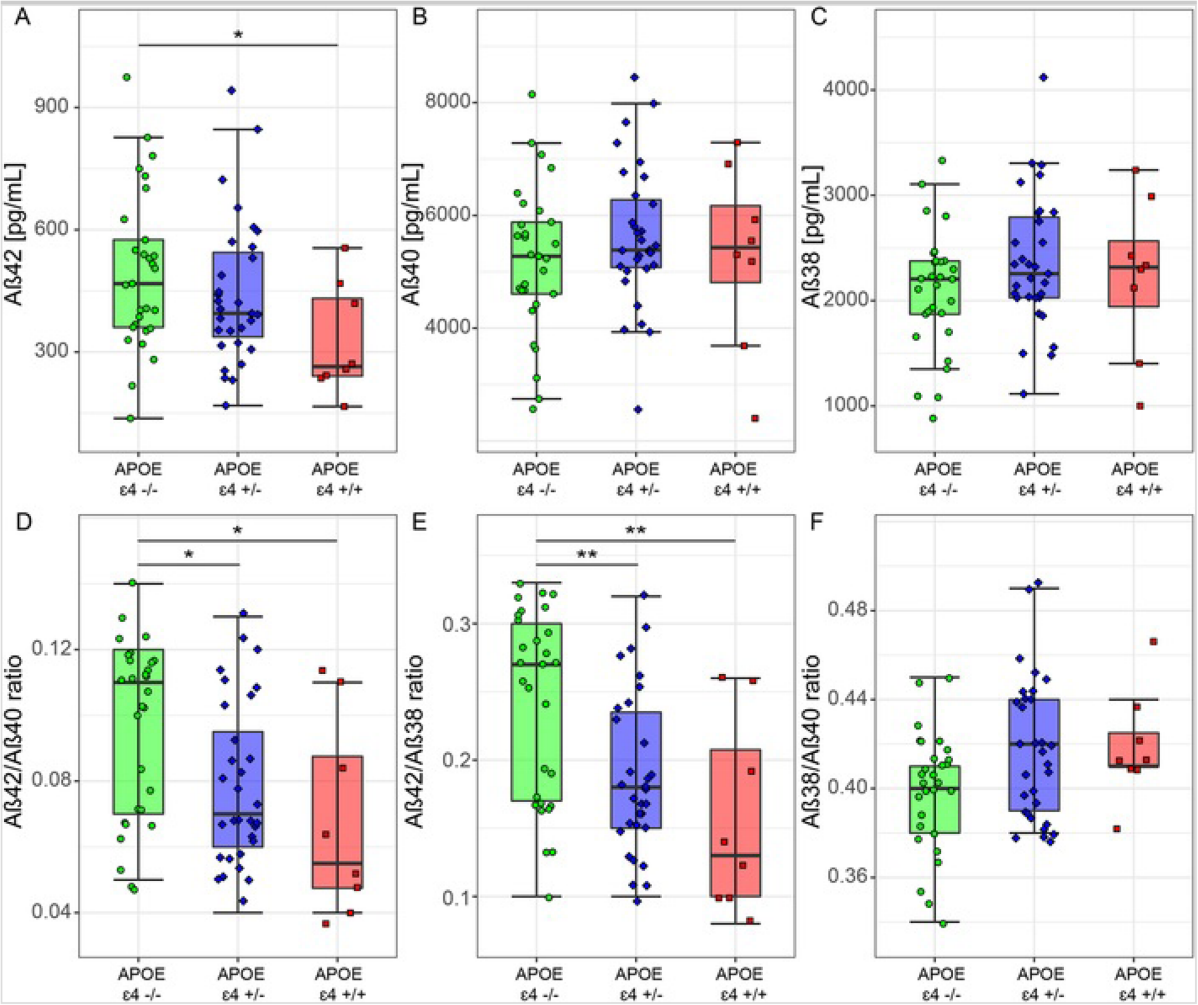
CSF biomarker levels by APOE genotype in DLB. **a** CSF levels of Aβ42. **b** CSF levels of Aβ40. **c** CSF levels of Aβ38. **d** CSF Aβ42/Aβ40 ratio. **e** CSF Aβ42/Aβ38 ratio. **f** CSF Aβ38/Aβ40 ratio. The line through the middle of the boxes corresponds to the median and the lower and the upper lines to the 25th and 75th percentile, respectively. The whiskers extend from the 5th percentile on the bottom to the 95th percentile on the top. Differences between groups were assessed with ANOVA corrected for age and sex and with a FDR multiple comparison correction. *p < 0.05, **p < 0.01, ***p < 0.001

To determine whether the observed differences in CSF Aβ peptides were influenced by the presence of co-morbid AD pathology, we analyzed CSF Aβ peptide levels in DLB patients with a CSF profile compatible with AD (DLB AD+, *n* = 23) and in DLB patients with a normal CSF profile (DLB AD−, *n* = 48). DLB AD+ patients were older and were more often APOE ε4 carrier than DLB AD− patients (Additional file 1: Table S2). CSF Aβ42 levels were lower in the DLB AD+ group compared to the DLB AD− group (*p* < 0.001, Fig. 3a) as was to be expected. There were no differences in levels of CSF Aβ40 and CSF Aβ38 between DLB AD+ and DLB AD− patients (*p* > 0.05, Fig. 3b, c). Furthermore, the Aβ42/Aβ40 and Aβ42/Aβ38 ratios were lower in the DLB AD+ group compared to the DLB AD− group (*p* < 0.001, Fig. 3d, e), while no difference was found for the Aβ38/Aβ40 ratio (*p* > 0.05, Fig. 3f). Next, we investigated whether APOE genotype influences CSF levels of Aβ peptides in DLB patients. CSF levels of Aβ42 were lower in DLB patients carrying two APOE ε4 alleles than in non-carriers (*p* < 0.05, Fig. 4a), and the Aβ42/Aβ40 and Aβ42/Aβ38 ratios were lower in APOE ε4 carriers compared to non-carriers in a gene dose-dependent manner (*p* < 0.05, Fig. 4d, e). In contrast, CSF levels of Aβ40, Aβ38, and Aβ38/Aβ40 ratios were similar in all APOE subgroups and did not show dose-dependent differences (Fig. 4b, c, f).

### CSF Aβ peptides and cognitive decline

**Fig. 5:**
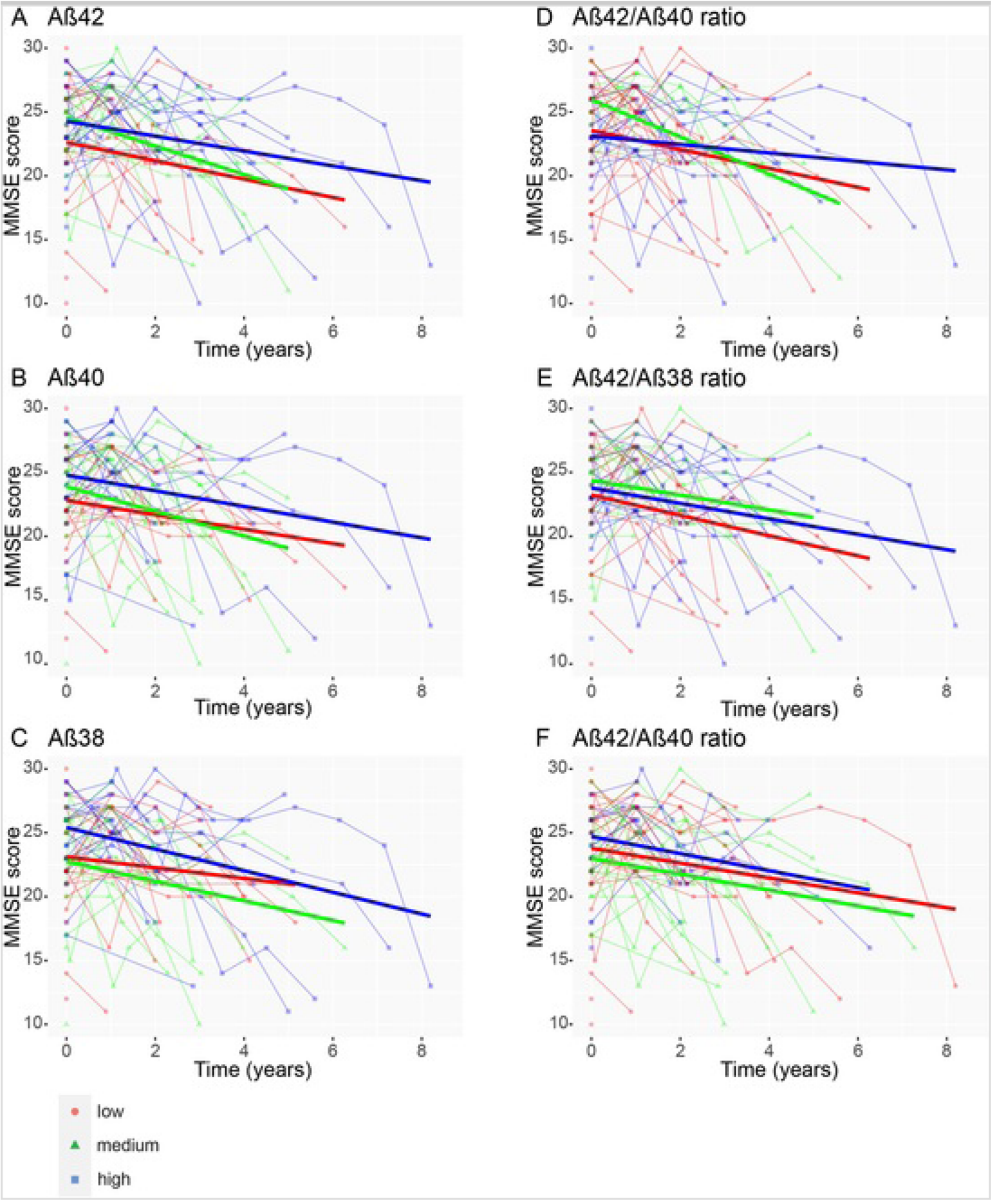
CSF Aβ peptide levels and cognitive decline in DLB. **a** Associations between baseline CSF Aβ42 levels and subsequent cognitive decline in the DLB group (*n* = 72) as measured by Mini-Mental State Examination (MMSE) score. **b** CSF Aβ40. **c** CSF Aβ38. **d** CSF Aβ42/Aβ40 ratio. **e** CSF Aβ42/Aβ38 ratio. **f** CSF Aβ38/Aβ40 ratio. Associations are shown using linear regression lines, with all DLB patients classified into tertile groups (low, medium, high) according to their CSF Aβ peptide levels for visualization purposes, but for the linear mixed model statistical analysis, continuous CSF Aβ peptide levels were used. Results were essentially the same when using Aβ peptide level tertiles as a categorical predictor

Table 2 and Fig. 5 demonstrate the results of linear mixed-effects models, which we used to test associations between CSF Aβ peptide levels and cognitive decline as examined by longitudinal change in MMSE score in DLB patients, adjusted for age, sex, and education. Lower levels of CSF Aβ42, CSF Aβ40, and CSF Aβ38 were associated with lower baseline MMSE scores (Aβ42: *β* = 1.02, SE = 0.45, *p* < 0.05; Aβ40: *β* = 1.11, SE = 0.43, *p* < 0.05; Aβ38: *β* = 1.03, SE = 0.43, *p* < 0.05), but CSF Aβ peptide levels were not associated with cognitive decline over time (interaction effect CSF Aβ peptide level × time, *p* > 0.05). In addition, no associations between Aβ peptide ratios and MMSE scores either at baseline or over time were found.

**Table 2:** Association of baseline CSF Aβ peptide levels with cognition over time in DLB

	MMSE score at baseline		Change in MMSE over time	
Predictors	*β* (SE)	*p*	*β* (SE)	*p*
Aβ42	1.02 (0.45)	0.027*	0.01 (0.19)	0.929
Aβ40	1.11 (0.43)	0.012*	− 0.13 (0.22)	0.551
Aβ38	1.03 (0.43)	0.020*	− 0.11 (0.22)	0.618
Aβ42/Aβ40 ratio	0.29 (0.49)	0.556	0.19 (0.20)	0.340
Aβ42/Aβ38 ratio	0.25 (0.48)	0.594	0.18 (0.20)	0.383
Aβ38/Aβ40 ratio	0.43 (0.44)	0.331	0.00 (0.23)	0.976

Data are presented as standardized estimates (*β*) with their standard error (SE) and *p* value. Linear mixed models were used with terms for time (years), CSF Aβ peptide measures, and an interaction term of CSF Aβ peptide measure × time as independent variables and MMSE score as the dependent variable. For all models, a random intercept and slope were assumed. Aβ peptide levels were transformed to *z*-scores. A beta coefficient of one (*β* = 1) therefore implies that a 1 standard deviation increase in CSF Aβ peptide was associated with a 1-point increase in MMSE score. The models (one model per CSF Aβ peptide) were adjusted for age, sex, and education

*Abbreviations*: *Aβ42* amyloid β_1-42_ determined with MSD ELISA assay, *Aβ40* amyloid β_1-40_ determined with MSD ELISA assay, *Aβ38* amyloid β_1-38_ determined with MSD ELISA assay

*p < 0.05

## Discussion

The main finding of this study is lower CSF levels of Aβ42, Aβ40, and Aβ38 peptides in a large group of DLB patients compared with controls, whereas AD patients presented with lower levels of CSF Aβ42 only, suggesting disease-specific aberrations in amyloid metabolism. Second, the observed differences in Aβ38 and Aβ40 were independent of co-morbid AD pathology and APOE genotype. Finally, low levels of all three CSF Aβ peptides were associated with more pronounced cognitive decline.

The finding of a selective drop of CSF Aβ42 in AD, whereas in DLB lower levels of CSF Aβ42 were accompanied by lower overall Aβ peptide levels, confirms previous observations by other groups [18–23]. These findings are also in line with a study in Parkinson’s disease (PD) showing that levels Aβ42, Aβ40 and Aβ38 were lower in CSF of early PD patients compared with controls [29]. The mechanisms underlying the different CSF Aβ peptide pattern in DLB compared to AD are unknown. An explanation could be that other non-AD-specific mechanisms affect global levels of all three Aβ peptides in the brain in DLB patients, since Aβ40 and Aβ38 levels in CSF are not expected to decrease as a result of AD pathology. To investigate this, we evaluated the effect of co-morbid AD pathology reflected by a CSF AD biomarker profile on CSF Aβ peptides in DLB. We found no differences in the levels of CSF Aβ40 and Aβ38 between DLB patients with co-morbid AD pathology and DLB patients without co-morbid AD pathology, suggesting that lower CSF levels of Aβ40 and Aβ38 levels in DLB were independent of co-morbid AD pathology. The finding of an association between CSF tau/Aβ42 and CSF Aβ38/Aβ40 in AD, whereas no association was observed in DLB, further supports the hypothesis that amyloid-β metabolism is different in DLB versus AD. Neither did we find an effect of APOE ε4 genotype on CSF Aβ40 and Aβ38 in DLB patients. A previous study reported an association between APOE ε4 genotype and reduced levels of CSF Aβ42 [30]. We observed only lower CSF Aβ42 levels in DLB patients carrying two APOE ε4 alleles. Our data seem to suggest that lower levels of all three Aβ peptides are likely due to DLB-specific mechanisms and that at least some of the mechanisms of action for APOE ε4 may be distinct from amyloidogenesis in DLB. Other researchers have similarly noted that APOE ε4 is associated with a greater severity of Lewy body pathology independent of co-morbid AD pathology [31]. Finally, we evaluated the effect of CSF Aβ peptides on cognitive decline in DLB patients. Low levels of CSF Aβ peptides were associated with lower MMSE scores at baseline, while low levels of CSF Aβ peptides were not associated with a steeper rate of cognitive decline over time. This is consistent with previous studies that showed CSF Aβ42 levels are inversely associated with cognitive function in DLB [23, 32]. Other studies have also demonstrated that co-morbid AD pathology was associated with more rapid cognitive decline over time in DLB [5, 8, 9].

Overall, our results might suggest that different pathogenic biological processes are involved in Aβ peptide-related amyloidogenesis in DLB versus AD. In AD, increased production and/or failure of clearance of Aβ42 lead to Aβ aggregation [11, 12] and Aβ aggregation subsequently result in low Aβ42 levels in CSF. DLB patients, however, showed in addition to low CSF Aβ42 levels also lower levels of CSF Aβ40 and Aβ38. The mechanism underlying the lower levels of all three Aβ peptides are likely related to dysregulation in amyloid precursor protein (APP) pathways. It is important to note that studies investigating APP processing in DLB are limited and therefore it is only possible to speculate about the explanations for our findings. APP processing is a complex process, and many factors are involved in the post-translational cleavage of APP into Aβ peptides. Cleavage of APP by α-secretase produces APPα, whereas cleavage by β-secretase generates APPβ and a C-terminal fragment (C99), which subsequently can be further metabolized by γ-secretase to produce Aβ peptides [11, 12]. Preclinical studies suggest that lower neuronal activity leads to reduced APP processing and consequently influences levels of Aβ peptides in the brain [33, 34]. In EEG studies, DLB patients showed marked slow-wave activity and more pronounced abnormalities compared with AD patients suggesting that reduced neuronal activity is prominent in DLB [35, 36]. In addition, synaptic dysfunction and, as a consequence, neurotransmitter deprivation and reduced neuronal activity could be directly linked to α-synuclein aggregates at synapses [37]. More direct evidence supporting the hypothesis of decreased APP processing in DLB is provided by a recent study that showed lower CSF levels of APPα and APPβ in DLB compared with AD and healthy controls [38]. Thus, the reduced activity in neuronal networks in DLB might result in diminished production of all Aβ peptides, including Aβ42. These results highlight the need for further studies into APP processing and Aβ accumulation in DLB.

Among the strengths of our study was the relatively large group (*n* = 72) of probable DLB patients, which were compared to age- and sex-matched AD patients and controls. Deep clinical phenotyping and clinical follow-up of all patients and controls were available. Furthermore, the etiological diagnosis was either supported by biomarkers or confirmed by autopsy. We therefore consider it unlikely that our results are significantly biased by clinical misdiagnosis. Controls included in the present study had normal CSF AD biomarkers at baseline and preserved normal cognitive function on neuropsychological testing. Using these inclusion criteria, we could almost certainly rule out AD. However, we were not able to exclude other causes of dementia since no reliable biomarkers are available yet. Another limitation is that data on Aβ peptide levels in CSF from patients with PD and PD patients with cognitive impairment were not available for the present study. Therefore, it is not possible at this time to incorporate the results into the full spectrum of Lewy body disease. Finally, we used the ratio of CSF tau/Aβ42 ≥ 0.52 as a surrogate measure for co-morbid AD pathology. It would be interesting to investigate the putative relevance of the novel AT(N) framework as a classification system for co-morbid AD pathology in DLB [39].

## Conclusion

Our results strongly suggest the presence of distinct CSF Aβ peptide profiles in DLB and AD, suggesting disease-specific aberrations in amyloid metabolism. In particular, the isolated drop of CSF Aβ42 in AD is likely a consequence of aggregation and deposition of Aβ in the brain. In contrast, DLB comes with reductions in all three CSF Aβ peptides, independent of co-morbid AD pathology or APOE genotype. These findings suggest that Aβ metabolism is affected in DLB, even in the absence of co-morbid AD pathology. Studies to elucidate the link between α-synuclein pathology and Aβ metabolism are vital to the understanding of Aβ peptide-related amyloidogenesis in DLB and could lead to novel therapeutic approaches.

## Supplementary information

**Additional file 1: Table S1.** Associations between Aβ peptides in CSF. **Table S2.** Demographics, clinical characteristics and CSF biomarker characteristics stratified by CSF. (DOCX 18 kb)

## Data Availability

The datasets used and/or analyzed during the current study are available from the corresponding author on reasonable request.

## Abbreviations

Aβ: Amyloid-β
AD: Alzheimer’s disease
APOE: Apolipoprotein E
APP: Amyloid precursor protein
CSF: Cerebrospinal fluid
DLB: Dementia with Lewy bodies
DAT-SPECT: (123) I-FP-CIT-SPECT scan
