# Supplementary material for: Amyloid-β peptides in cerebrospinal fluid of patients with dementia with Lewy bodies
Source: Alzheimers Res Ther. 2019 Oct 10;11:83. doi: 10.1186/s13195-019-0537-5 (PMC6788069; doi:10.1186/s13195-019-0537-5)
Supplement: Supplementary file 1 — Additional file 1: Table S1. Associations between Aβ peptides in CSF. Table S2. Demographics, clinical characteristics and CSF biomarker characteristics stratified by CSF. (DOCX 18 kb) [file 13195_2019_537_MOESM1_ESM.docx]

ADDITIONAL FILE 1:

**Table S1. Associations between Aβ peptides in CSF**

|  | Aβ42 | Aβ40 | Aβ38 | Aβ42/Aβ40 ratio | Aβ42/Aβ38 ratio | Aβ38/Aβ40 ratio | Tau/Aβ42 ratio |
| --- | --- | --- | --- | --- | --- | --- | --- |
| **Total (n=148)** |  |  |  |  |  |  |  |
| Age | -0.202 | 0.034 | 0.041 | -0.259 | -0.242 | 0.022 | 0.094 |
| Sex | 0.088 | 0.161 | 0.183 | 0.007 | -0.029 | 0.188 | 0.072 |
| Aβ42 | - | 0.568*** | 0.530*** | 0.805*** | 0.741*** | 0.132 | -0.556*** |
| Aβ40 | 0.568*** | - | 0.967*** | 0.011 | -0.067 | 0.392*** | 0.124 |
| Aβ38 | 0.530*** | 0.967*** | - | -0.013 | -0.129 | 0.605*** | 0.179 |
| Aβ42/Aβ40 ratio | 0.805*** | 0.011 | -0.013 | - | 0.973*** | -0.116 | -0.759*** |
| Aβ42/Aβ38 ratio | 0.741*** | -0.067 | -0.129 | 0.973*** | - | -0.304** | -0.776*** |
| Aβ38/Aβ40 ratio | 0.132 | 0.392*** | 0.605*** | -0.116 | -0.304** | - | 0.292** |
| Tau/Aβ42 ratio | -0.556*** | 0.124 | 0.179 | -0.759*** | -0.776*** | 0.292** | - |
| **DLB (n=72)** |  |  |  |  |  |  |  |
| Aβ42 | - | 0.546*** | 0.501*** | 0.746*** | 0.676*** | 0.095 | -0.450** |
| Aβ40 | 0.546*** | - | 0.969*** | -0.107 | -0.181 | 0.406** | 0.286 |
| Aβ38 | 0.501*** | 0.969*** | - | -0.135 | -0.248 | 0.609*** | 0.309 |
| Aβ42/Aβ40 ratio | 0.746*** | -0.107 | -0.136 | - | 0.970*** | -0.205 | -0.711*** |
| Aβ42/Aβ38 ratio | 0.676*** | -0.181 | -0.248 | 0.970*** | - | -0.400** | -0.719*** |
| Aβ38/Aβ40 ratio | 0.095 | 0.406* | 0.609*** | -0.205 | -0.400* | - | 0.257 |
| Tau/Aβ42 ratio | -0.450** | 0.286 | 0.309 | -0.711*** | -0.719*** | 0.257 | - |
| **AD (n=38)** |  |  |  |  |  |  |  |
| Aβ42 | - | 0.662*** | 0.534* | 0.569** | 0.513* | -0.075 | -0.295 |
| Aβ40 | 0.662*** | - | 0.927*** | -0.178 | -0.229 | 0.237 | 0.115 |
| Aβ38 | 0.534* | 0.927*** | - | -0.251 | -0.438 | 0.579** | 0.288 |
| Aβ42/Aβ40 ratio | 0.569** | -0.178 | -0.251 | - | 0.853*** | -0.303 | -0.495* |
| Aβ42/Aβ38 ratio | 0.513* | -0.229 | -0.438 | 0.853*** | - | -0.674*** | -0.600** |
| Aβ38/Aβ40 ratio | -0.075 | 0.237 | 0.579** | -0.303 | -0.674*** | - | 0.521 |
| Tau/Aβ42 ratio | -0.295 | 0.115 | 0.288 | -0.495* | -0.600** | 0.521* | - |
| **Controls (n=38)** |  |  |  |  |  |  |  |
| Aβ42 | - | 0.844*** | 0.836*** | 0.588** | 0.415 | 0.408 | 0.293 |
| Aβ40 | 0.844*** | - | 0.982*** | 0.092 | -0.081 | 0.441 | 0.558** |
| Aβ38 | 0.836*** | 0.982*** | - | 0.105 | -0.126 | 0.591** | 0.561** |
| Aβ42/Aβ40 ratio | 0.588** | 0.092 | 0.105 | - | 0.903*** | 0.122 | -0.280 |
| Aβ42/Aβ38 ratio | 0.415 | -0.081 | -0.126 | 0.903*** | - | -0.289 | -0.399 |
| Aβ38/Aβ40 ratio | 0.408 | 0.441 | 0.591** | 0.122 | -0.289 | - | 0.310 |
| Tau/Aβ42 ratio | 0.293 | 0.558** | 0.561** | -0.280 | -0.399 | 0.310 | - |

Associations were assessed with Pearson correlations. FDR corrections were used to adjust *p* values for multiple comparisons. ** p<0.05, ** p<0.01, *** p<0.001*.

**Table S2. Demographics, clinical characteristics and CSF biomarker characteristics stratified by CSF tau/Aβ42 ratio in DLB**

|  | **DLB AD- (n=48)** | **DLB AD+ (n=23)** |
| --- | --- | --- |
| Female (n, %) | 4 (8%) | 2 (8%) |
| Age (mean ± SD) | 66 ± 6^a^ | 70 ± 5 |
| MMSE (median [IQR]) | 23 [22-26] | 23 [19-26] |
| APOEε4 carrier (n, %) | 23 (47%)^a^ | 16 (80%) |
| CSF AD biomarkers Innotest (median [IQR]) |  |  |
| Aβ_1-42_(pg/ml)* | 964 [778-964]^b^ | 622 [524-666] |
| t-tau (pg/ml) | 279 [216-311]^b^ | 449 [358-634] |
| p-tau (pg/ml) | 44 [31-49]^b^ | 66 [53-77] |
| CSF Aβ peptides  MSD (mean ± SD) |  |  |
| Aβ42 (pg/ml) | 510 ± 181^b^ | 309 ± 103 |
| Aβ40 (pg/ml) | 5314 ± 1354 | 5716 ± 1316 |
| Aβ38 (pg/ml) | 2185 ± 652 | 2393 ± 607 |
| Aβ42/Aβ40 ratio | 0.10 ± 0.02^b^ | 0.05 ± 0.01 |
| Aβ42/Aβ38 ratio | 0.24 ± 0.06^b^ | 0.13 ± 0.03 |
| Aβ38/Aβ40 ratio | 0.41 ± 0.03 | 0.42 ± 0.03 |

*Data are presented as mean ± SD, median [interquartile range] or n (%).
Differences between groups were assessed with t-tests, χ^2^, and Mann-Whitney U tests were performed where appropriate. For CSF Aβ peptides, differences between diagnostic groups were assessed using ANOVA corrected for age and sex.*

*a p<0.05 compared to DLB AD+
b p<0.001 compared to DLB AD+
*Levels of Innotest Aβ_1-42_ were drift corrected (Tijms et al., Clin Chem 2018)
Abbreviations: Aβ42* = *amyloid β_1-42_ determined with MSD ELISA assay; Aβ40 = amyloid β_1-40_ determined with MSD ELISA assay; Aβ38 = amyloid β_1-38_ determined with MSD ELISA assay; DLB = dementia with Lewy bodies; MMSE = mini-mental state examination; MSD = Meso Scale Discovery.*
